# 

**Published:** 1883-01

**Authors:** 


### Pamphlets.

Conjoint session of the North Carolina Board of Health and Medical Society of North Carolina, held in Concord, May 10, 1882.

Annual Report of the National Board of Health for the year 1882.

Feeding per Rectum: as illustrated in the case of President Garfield and others. By D. W. Bliss, M.D., Washington, D. C.

Some thoughts on Phthisis, with special reference to Laryngeal Symptoms in Diagnosis. By M. F. Coomes, M.D., Louisville, Ky. Reprint from the *Archives of Laryngology*.

Menstrual Amblyopia. By M. F. Coomes, M.D., Louisville, Ky. Reprint from the *Medical Herald.*

Excision of Cartilage in Nasal Obstruction due to deviated septum. By John B. Roberts, M.D., Philadelphia. Reprint from the *Transactions of the Pennsylvania State Medical Society.*

